# Steroid hormones in Pacific walrus bones collected over three millennia indicate physiological responses to changes in estimated population size and the environment

**DOI:** 10.1093/conphys/coaa135

**Published:** 2021-01-19

**Authors:** Patrick Charapata, Lara Horstmann, Nicole Misarti

**Affiliations:** 1 College of Fisheries and Ocean Sciences, University of Alaska Fairbanks, PO Box 757220, Fairbanks, AK 99775, USA; 2 Water and Environmental Research Center, University of Alaska Fairbanks, PO Box 755910, Fairbanks, AK 99775, USA; 3 Department of Biology, Baylor University, One Bear Place, Waco, TX 76706, USA

**Keywords:** Bone steroid hormones, Pacific walrus, Arctic, climate change, stress biomarker, reproductive status, population estimates

## Abstract

The Pacific walrus (*Odobenus rosmarus divergens*) is an iconic Arctic marine mammal and an important resource to many Alaska Natives. A decrease in sea ice habitat and unknown population numbers has led to concern of the long-term future health of the walrus population. There is currently no clear understanding of how walrus physiology might be affected by a changing Arctic ecosystem. In this study, steroid hormone concentrations (progesterone, testosterone, cortisol and estradiol) were analysed in walrus bones collected during archaeological [3585–200 calendar years before present (BP)], historical [1880–2006 common era (CE)] and modern (2014–2016 CE) time periods, representing ~ 3651 years, to track changes in reproductive activity and cortisol concentrations (biomarker of stress) over time. Our results show that modern walrus samples have similar cortisol concentrations (median = 43.97 ± standard deviation 904.38 ng/g lipid) to archaeological walruses (38.94 ± 296.17 ng/g lipid, *P* = 0.75). Cortisol concentrations were weakly correlated with a 15-year average September Chukchi Sea ice cover (*P* = 0.002, 0.02, *r*^2^ = 0.09, 0.04, for females and males, respectively), indicating a possible physiological resiliency to sea ice recession in the Arctic. All steroid hormones had significant negative correlations with mean walrus population estimates from 1960 to 2016 (*P* < 0.001). Progesterone in females and testosterone in males exhibited significant correlations with average September Chukchi Sea ice cover for years 1880–2016 (*P* < 0.001 for both, *r^2^* = 0.34, 0.22, respectively). Modern walruses had significantly lower (*P* = < 0.001) reproductive hormone concentrations compared with historic walruses during times of rapid population increase, indicative of a population possibly at carrying capacity. This is the first study to apply bone as a tool to monitor long-term changes in hormones that may be associated with changes in walrus population size and sea ice cover.

## Teaser

Reproductive and stress-related hormones were measured in Pacific walrus bones collected over three millennia to understand the effects current climate change has on walrus physiology. Bone steroid hormones significantly changed over time and correlated with sea ice extent and population estimates, providing evidence that they are biomarkers of walrus physiology.

## Introduction

The Pacific walrus (*Odobenus rosmarus divergens*, hereafter referred to as walrus) is an important Arctic marine mammal that Russian and Alaskan Natives rely on for cultural, economic and subsistence purposes (Metcalf and Robards, 2008). Walruses are benthic predators foraging on bivalves, gastropods and marine worms as their preferred prey (Fay, 1982; Sheffield and Grebmeier, 2009), although feeding on higher trophic level prey, such as seals and sea birds, has been documented (Lowry and Fay, 1984; Seymour *et al.*, 2014). Females, calves and some male walruses use summer ice in the Chukchi Sea as a feeding, resting and molting platform (Fay, 1982). Walruses passively float on sea ice to different benthic feeding locations in the Chukchi Sea conserving energy that can be used for other purposes (e.g. energy stores and/or reproduction; Fay, 1982; Jay *et al.*, 2012).

Recently, Arctic warming has caused sea ice to recede into deep Arctic basin waters, which may limit access to benthic prey due to walruses’ relatively shallow dive limit of ~250 m (Fay and Burns, 1988; Jay and Fischbach, 2008; Jay *et al.*, 2012). Walruses have responded to this altered summer habitat by using coastal land haulouts, where they may feed in less productive areas or go on extensive, and energetically costly, foraging trips (up to 200 km one way) to Hanna Shoal (Jay *et al.*, 2012). Females are most affected by the loss of access to rich foraging grounds due to the higher energy requirements needed for reproduction (Noren *et al.*, 2014). The Chukchi Sea remains a rich benthic foraging ground, but northerly shifts of walruses and their prey in the Northern Bering Sea are predicted due to a potential change to a more pelagic food web (Grebmeier *et al.*, 2006; Grebmeier, 2012; Jay *et al.*, 2014; Schonberg *et al.*, 2014). However, higher than expected phytoplankton productivity may continue to sustain the benthos (Arrigo *et al.*, 2012, 2014; Ardyna and Arrigo, 2020).

A change in the movement patterns of walruses in response to receding sea ice has already caused changes to Alaska Native hunting patterns (Fidel *et al.*, 2014). For example, hunters must undergo longer and more dangerous hunting excursions to pursue walruses (Fidel *et al.*, 2014). In addition, researchers from Wrangel Island reported more malnourished females compared to years with adequate sea ice (Metcalf and Robards, 2008). However, more recently, hunters from St. Lawrence Island have reported the majority of walruses in good body condition (Quakenbush *et al.*, 2016). According to local hunters in Utqiaġvik (formerly Barrow), the hunting season for walruses has become shorter and more variable due to the loss of sea ice (Huntington *et al.*, 2015). While the Pacific walrus has been removed as a candidate for the endangered species list (MacCracken *et al.*, 2017), human dependency on walruses reflects the importance of understanding the long-term health of the walrus population in response to loss of sea ice habitat.

One way to understand the health of a remote wild population is through physiological studies, including collecting tissues and analysing how hormone concentrations have changed throughout a specified time period. For walruses, understanding how reproductive and stress-related hormone concentrations vary in response to reduced sea ice could give insight into the resiliency of the walrus population relative to climate change. Monitoring changes in steroid hormones in walrus bone over periods encompassing environmental change could detect a shift in the physiological baseline of walruses in response to reduced sea ice (Charapata *et al.*, 2018). The modern walrus population is not ideal for studying the physiological resilience (i.e. changes in reproductive and stress biomarker hormone concentrations) to receding sea ice because a significant reduction of sea ice over the Chukchi Sea’s shelf has occurred over at least the past 13 years (Jay *et al.*, 2012). Thus, monitoring acute stress and reproductive responses with serum and blubber hormone studies of only current and future walrus populations would be an inaccurate portrayal of their physiological resilience to climate change in the Arctic. The physiological status (i.e. concentrations of reproductive and stress biomarker hormones) of archaeological and historic walruses would provide a more precise picture of the degree to which a population is affected by the recent drastic reductions in sea ice.

Over the archaeological time period related to this study [3585–200 years before present (BP), note; 200 BP = 1750 common era (CE)], the Arctic climate was significantly different from the industrial historic and modern Arctic climate (1750 CE–present; IPCC, 2013; Clark *et al.*, 2019). In general, the pre-industrial climate in the Arctic was undergoing a Neoglacial cooling period from 4000 to 3000 BP, followed by natural warming events (e.g. Medieval and Roman Warming periods) before entering the Little Ice Age (430–230 BP) (de Vernal *et al.*, 2005; Kaufman *et al.*, 2016; Clark *et al.*, 2019). Recently, (1950 CE–present), there has been a reversal in an Arctic cooling trend attributed in part to anthropogenic greenhouse emissions (Kaufman *et al.*, 2009). The possible advection of unprecedented warm Atlantic water into the Arctic is a factor in the rapid sea ice decreases observed today (Spielhagen *et al.*, 2011). Thus, today’s walrus population has to adjust to changes occurring more rapidly than past populations. Walruses from archaeological time periods can be used as a ‘physiological baseline’ of sorts to compare with the physiology of present-day walruses.

Steroid hormones are a useful tool for understanding reproductive and stress-related physiological changes in pinnipeds. Typically in female pinnipeds, estradiol concentrations increase during estrus, and progesterone increases during ovulation and pregnancy (Boyd, 1991; Pomeroy, 2011). For male pinnipeds, testosterone induces seminiferous tubules and epididymis growth and has been associated with stimulating spermatogenesis in male walruses (Pomeroy, 2011; Muraco *et al.*, 2012). Cortisol has a suite of physiological effects outside of its role in the hypothalamus–pituitary–adrenal axis (MacDougall-Shackleton *et al.*, 2019), but it is a relevant stress biomarker for pinnipeds that increases during natural (molting and reproduction; Kershaw and Hall, 2016) and life-threatening chronic (strandings, Beaulieu-McCoy *et al.*, 2017) stressors. These steroid hormones (progesterone, testosterone, cortisol and estradiol) have been measured in cortical bone of walruses collected over 3000 years ago with long estimated reservoir times (~15 years for progesterone, testosterone and cortisol and ~1 year for estradiol) (Charapata *et al.*, 2018) and in modern terrestrial mammal bone (Yarrow *et al.*, 2010). However, bone steroid hormones have not been utilized to monitor long-term reproductive and stress-related hormone changes in any Arctic marine mammals in response to rapid changes in the Arctic.

In this study, steroid hormone concentrations from archaeological (3585–200 BP), historical (1880–2006 CE) and modern (2014–2016 CE) walrus bone were analysed to investigate possible changes in reproductive status (i.e. changes in estradiol, progesterone and testosterone) and cortisol concentrations (biomarker of stress) of walruses. This study (i) compared baseline cortisol concentrations of archaeological bone with modern and historic bone cortisol concentrations as well as finer decadal timescales, (ii) determined if bone steroid hormones were correlated with changes in walrus population size and minimum sea ice cover through time and (iii) determined if bone reproductive hormones reflect the reproductive activity of modern walruses. A secondary objective of this study was to determine if the sex of unknown individuals could be determined using steroid hormone concentrations.

## Materials and methods

### Sample collection

Walrus bone samples (*n* = 281) were collected and categorized into time periods, including archaeological (3585–200 calendar years BP, *n* = 53; Supplementary S1), historical (1880–2006 CE, *n* = 155; Supplementary S2) and modern (2014–2016 CE, *n* = 73; Supplementary S3). The archaeological time period was determined from the oldest bone in the study (3585 BP) to the relative start of the industrial revolution (200 BP or 1750 CE; IPCC, 2013). The historical time period was based on the oldest and newest bones found in marine mammal collections of the University of Alaska Museum (UAM) and the Smithsonian Institution National Museum of Natural History (1880–2006 CE). There is a potential 70-year overlap in archaeological and historical time periods (1880–1950 CE, or 70–0 BP; Supplementary S1 and S2). However, the majority of archaeological samples (see Supplementary S1) fall outside this overlapping time period and only 24 of the 155 historical samples potentially overlap with archaeological time periods (Supplementary S1 and S2). Additionally, this same overlap in sample collection dates was present in Clark *et al.* (2019) but did not significantly affect analyses of stable carbon and nitrogen isotope ratios in walrus bone. The modern time period was based on the sampling of walruses from Alaska Native hunters during 2014–2016 CE. Bones from historical and modern time periods were further divided into shorter decadal time scales where ecological walrus data were available for improved interpretation and understanding of bone steroid hormone concentrations and to put into context modern walrus hormone concentrations. This includes the 1880s (*n* = 5), 1890s (*n* = 2), 1900s (*n* = 1), 1910s (*n* = 1), 1920s (*n* = 2), 1930s (*n* = 13), 1950s (*n* = 31), 1960s (*n* = 36), 1970s (*n* = 27), 1980s (*n* = 25), 1990s (*n* = 10), 2000s (*n* = 2) and 2010s (*n* = 73). Hormones measured in paired walrus skull and mandible samples were similar (Charapata *et al.*, 2018). We therefore assumed hormones measured in cortical bone among different walrus elements were comparable (Supplementary S1, S2, and S3). Total samples included 94 females, 127 males and 60 walruses of unknown sex.

### Archaeological samples

**Figure 1:**
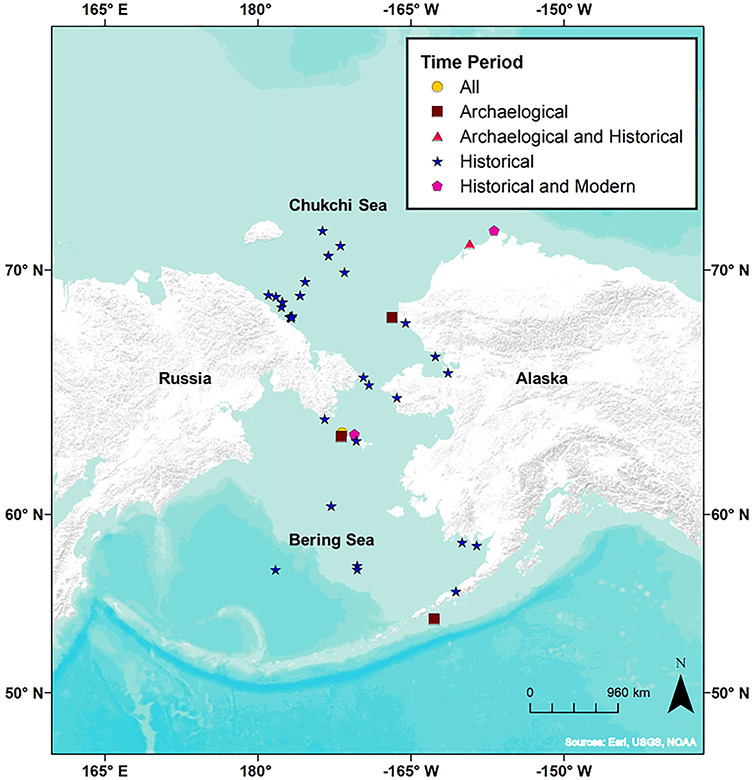
Sampling locations of archaeological, historical and modern Pacific walrus bone from Alaska and Russia. ‘All’ represents locations where walrus bones were collected from all three time periods (orange circles); ‘Archaeological’ represents locations where only archaeological bone was collected (brown squares); ‘Archaeological and Historical’ indicate locations where walrus bones from both archaeological and historical time periods were collected (pink triangles); ‘Historical’ represents locations where walrus bones from only the historical time period were collected (blue stars); and ‘Historical and Modern’ represents locations where walruses from both historical and modern time periods were collected for this study (magenta pentagons). Each walrus sample location corresponds to locations in the Supplementary S1-S3. The only sampling locations not depicted on the map are ‘South Chukchi Sea’, ‘Unknown’, ‘Bristol Bay’, ‘Bering Sea’ and ‘West Chukchi Sea’, as they do not have a specific latitude/longitude.

Archaeological walrus bones were acquired from numerous archaeological sites throughout the range of walruses in Alaska and Russia (Fig. 1, Supplementary S1). Walrus bones from multiple sites across Alaska were obtained through the UAM Archaeological Collection and through the Ukpeaġvik Iñupiat Corporation in Utqiaġvik, Alaska. The minimum number of individuals was determined by selecting the largest number of the most common bone element located in each site or separate unit and depth within a site to ensure that each bone sampled represented one individual (Grayson, 1978, 1984; Supplementary S1). Walrus bones, including historical bones (discussed below), were assigned an estimated age class (e.g. subadult and adult) based on a combination of size and degree of fusion between the respective element and its epiphyses when possible (Davis, 1992). If an archaeological bone was fragmented, it was not assigned an age class. Calibrated radiocarbon ages were acquired from Clark *et al.* (2019) and archaeological site reports (Supplementary S1). Sex of archaeological samples (*n* = 6) was determined by morphometric differences between skulls of males and females (Taylor *et al.*, 2020). The sexes of the remaining samples were unknown (*n* = 47).

### Historical samples

Historical samples (*n* = 155) were acquired from the UAM Mammalogy Collection and the Smithsonian Institution National Museum of Natural History. Only samples with a collection date and location based on museum records were used. Any additional data including age class, sex, pregnancy status and presence of offspring come from museum records curated by their respective sources. A list of samples with the respective provenience data is provided in Supplementary S2.

### Modern samples

Modern samples were collected from Native subsistence harvests through an agreement with Native hunters, the Eskimo Walrus Commission, the Alaska Department of Fish and Game and the US Fish and Wildlife Service (USFWS) during April and May 2014–2016. Hunters recorded sex, age class for all walruses and reproductive information for harvested females, including pregnancy (fetus presence/absence), lactation and whether they were accompanied by calves and/or yearlings. Bone samples were transferred to Dr L. Horstmann at the University of Alaska Fairbanks for sample analysis under a letter of authorization from the USFWS. Utqiaġvik in partnership with the North Slope Borough Department of Wildlife Management and subsistence hunters from the Utqiaġvik area provided additional samples (*n* = 3). A list of modern samples with provenience data (e.g. sex and age class) is provided in Supplementary S3.

### Walrus population estimates

Steroid hormone concentrations measured in walrus bone collected during 1960–2016 were correlated with minimum, mean and maximum population estimates for the Pacific walrus population. An estimated mean range of 135 359 walruses was calculated using the ranges from published population numbers corrected for undetected walruses underwater from Table 3 in Fay *et al.* (1997) and the range reported in Speckman *et al.* (2011). This range was applied to population estimates that did not have a mean or maximum population size (Gilbert *et al.*, 1992; Fay *et al.*, 1997; MacCracken *et al.*, 2017) for correlation analysis with steroid hormone concentrations. This was done to consider the variability among population estimates calculated using different methods and area coverage. To determine walrus population growth rates for years without a published estimate, we used a geometric growth rate model used in walrus population projections (Udevitz *et al.*, 2013):

*GR = N_i_/N_j_^1/(i-j)^*

where GR = approximated growth rate, *N*_i_ = population from year i, where year i > year j, and *N*_j_ = population from year j (Udevitz *et al.*, 2013). For years without an estimate, we then applied published accounts of the minimum, mean and maximum walrus population (Supplementary S4) to the following formula:

P_i_ = P_j_*GR

where *P*_i_ = walrus population at year i and *P*_j_ = walrus population the previous year in relation to P_i_ and GR = the geometric growth rate of the walrus population (Udevitz *et al.*, 2013). Population estimates during 1880–1959 were only used to visualize walrus population trends. Approximated walrus populations derived from peer-reviewed studies during 1960–2016 were only used to determine any potential correlations between walrus population changes and steroid hormone concentrations.

### Steroid hormone analysis

We followed the methods for extracting and measuring steroid hormones in walrus bone previously published in Charapata *et al.* (2018). Briefly, pieces of cortical bone were pulverized into a powder. Subsequently, methanol (5:1 methanol: bone powder ratio) and 100 ng of isotopically labelled internal standards were added to bone powder. Samples were then homogenized for 3 minutes and set on a rocking platform for 24 hours. Samples were then centrifuged with supernatant collected and dried using nitrogen gas. Samples were shipped to Purdue University, IN, where steroid hormone concentrations were measured using liquid chromatography/tandem mass spectrometry (LC/MS/MS) analysis. Raw concentrations (ng) from LC/MS/MS analysis were then corrected using an average percent lipid correction factor (4.83%, 1.98% and 2.71% for modern, historical and archaeological walrus bones, respectively) based on a modified 2:1 chloroform: methanol Soxhlet lipid extraction of walrus bones collected from the different time periods (*n* = 12, 10 and 12 for modern, historical and archaeological, respectively, see Charapata *et al.*, 2018). Thus, all hormone concentrations are presented in units of ng/g lipid. Any samples with hormone concentrations below detectable limits were assigned a value of half the detection limit (detection limit of LC/MS/MS instrument is 0.50 ng, and half the detection limit of 0.25 ng was assigned to non-detectable (ND) samples; Charapata *et al.*, 2018). Detection limits were ~2.0 ng/g bone (non-lipid corrected) for progesterone and estradiol (Charapata *et al.*, 2018) and 0.04 ng/g bone and 0.16 ng/g bone for cortisol and testosterone, respectively. There were a total of *n* = 35, n = 0, *n* = 0 and *n* = 32 ND samples for progesterone, testosterone, cortisol and estradiol, respectively. The extraction efficiencies were 51%, 107%, 72% and 79% for progesterone, testosterone, cortisol and estradiol, respectively, and final concentrations were corrected by the known amount of isotopically labelled standard added to each sample (Charapata *et al.*, 2018).

### Statistical analysis of steroid hormones over time, with population estimates and sex assignment

Steroid hormone concentrations in walrus bone were not normally distributed; therefore, non-parametric tests were used for certain hormone analyses. Kruskal–Wallis analysis of variance (ANOVAs) was used to determine differences among archaeological, historical and modern periods (sample time periods), decades and between sexes (Anderson, 2001; Anderson and Walsh, 2013; Wang *et al.*, 2016). A Mann–Whitney pairwise comparison test was performed when the Kruskal–Wallis tests were significant using an alpha of 0.05. A Dunn’s test was used for decadal comparisons due to the numerous comparisons performed. The 1900s and 1910s were not included in the decadal comparisons due to low sample sizes (*n* = 1 for each decade). Similarity percentages (one-way SIMPER) analysing Bray–Curtis distances among samples were used to determine which hormone concentrations contributed to differences among different time periods (Mejri *et al.*, 2014). Correlations among steroid hormone concentrations in walrus bones (*n* = 173 total, *n* = 100 males, *n* = 68 females and *n* = 5 unknowns) and walrus population estimates throughout time (1960s–2010s) were analysed using Spearman’s rank correlation (Wilson *et al.*, 1997). Spearman rank correlations were also performed using hormones measured in only females (*n* = 68) and walrus population estimates during 1960s–2010s.

Log-transformed progesterone, testosterone and Box–Cox transformed cortisol data were used from known females and males to determine if hormone data could correctly classify the sex of walruses using linear discriminant analysis (LDA). A jackknife approach was used for each known sexed sample and run through the LDA to get an adjusted jackknife classification percentage.

### Statistical analysis of steroid hormones and Chukchi Sea ice

Annual estimates of September Chukchi Sea ice cover, expressed as a percentage, for years 1850 CE–present were extracted from the Scenarios Network for Alaska and Arctic Planning (SNAP) Sea Ice Atlas (http://www.snap.uaf.edu). Early sources (~1850 CE) for sea ice estimates in the SNAP Sea Ice Atlas come from analysis of whaling records (Mahoney *et al.*, 2011), while current sea ice estimates come from National Snow and Ice Data Center microwave sensor data (http://seaiceatlas.snap.uaf.edu/about). September sea ice cover was chosen because September is typically when the sea ice minimum is reached and walruses are present in the Chukchi Sea (see Clark *et al.*, 2019).

Progesterone, testosterone and estradiol data were log transformed, and cortisol data were Box–Cox transformed to achieve normality. These normalized data were then used for linear regressions of hormone data with September Chukchi Sea ice cover estimates. Log-transformed estradiol data did not completely achieve normal distributions. However, log-transformed data, large sample sizes (*n* = 122 males and *n* = 93 females) and simple linear regressions being robust to small deviations of normality (Noorossana *et al.*, 2011) allowed us to confidently perform a linear regression of annual Chukchi Sea September sea ice coverage and log-transformed estradiol concentrations. Walruses included in the linear regression analyses were comprised of known females and males with known collection dates from historical and modern time periods. A rolling 15-year average of percent sea ice cover of the Chukchi Sea in September was calculated for each walrus based on its collection year to account for steroid hormone reservoir time in walrus bone (Charapata *et al.*, 2018). Linear regressions were performed among the 15-year average of percent sea ice cover for the month of September in the Chukchi Sea with female and male progesterone, testosterone and cortisol data. The same analysis was performed with estradiol concentrations, but with yearly September average ice coverage, because estradiol has a shorter reservoir time in walrus cortical bone (Charapata *et al.*, 2018).

All statistical analyses were performed in PAST (V 3.20, Hammer *et al.*, 2001). An alpha of 0.05 was used for all analyses. All statistical differences among the steroid hormone concentrations are reported as medians; medians are robust to outliers. All data are reported as median ± 1 standard deviation (SD), with mean values given for reference. Standard errors (SEs) are used in figures for effective visualizations of trends in data.

## Results

### Steroid hormone concentrations from archaeological, historical, modern and finer decadal (1880s–2010s) time periods

Concentrations of all steroid hormones in this study were significantly different among sample time periods (Kruskal–Wallis ANOVAs, *P* = < 0.001 for progesterone, testosterone and estradiol, and *P* = 0.002, cortisol). Historical (1880–2006) samples had steroid hormone concentrations significantly higher than both modern (2014–2016, Mann–Whitney, *P* < 0.001, progesterone, testosterone and estradiol, and *P* = 0.002, cortisol) and archaeological samples (3585–200 BP, *P* < 0.001, progesterone, testosterone and estradiol and *P* = 0.01, cortisol; Table 1). Archaeological and modern samples had similar progesterone, testosterone, cortisol and estradiol concentrations (*P* = 0.38, 0.35, 0.75 and 0.06, respectively; Table 1). The contribution of each steroid hormone (SIMPER) to the differences among sample time periods is summarized in Table 2. Overall, differences in reproductive hormones, specifically progesterone and estradiol, contributed the most to the differences observed among sample time periods. Cortisol contributed < 10% to the differences among sample time periods. Steroid hormone concentrations were highly variable for all time periods (Table 1), which were driven by differences in estradiol and progesterone concentrations (SIMPER; Table 2).

**Table 1:** Mean and median steroid hormone concentrations (i.e. progesterone, testosterone, cortisol and estradiol) ± 1 SD, concentration ranges and sample sizes (*n*), for each walrus sample time period (i.e. archaeological, historical and modern)

Sample time period	Sample size *(n)*	Hormone	Mean ± 1 SD median (ng/g lipid)	Range: min–max (ng/g lipid)	Comparison	*P-*value
		Progesterone	368.27 ± 1,199.62 101.20	15.09–8,740.18		0.38
Archaeological (>200 BP)	53	Testosterone	259.10 ± 285.76 199.76	35.61–1,803.85	Modern	0.34
		Cortisol	125.48 ± 296.17 38.94	13.70–1,889.60		0.75
		Estradiol	1,600.07 ± 2146.16 59.22	10.01–7161.93		0.06
		Progesterone	10,399.90 ± 35,097.02 1,147.65	41.71–276,407.72		**<0.001**
Historical (1880–2006 CE)	155	Testosterone	1,870.00 ± 4,051.95 716.60	8.09–4,178.13	Archaeological	**<0.001**
		Cortisol	568.24 ± 1,496.78 69.98	2.24–1,0412.57		**0.01**
		Estradiol	3,954.20 ± 3,171.80 5,607.04	14.46–9,754.42		**<0.001**
		Progesterone	440.12 ± 845.23 112.38	3.49–5,464.69		**<0.001**
Modern (2014–2016 CE)	73	Testosterone	443.99 ± 1,677.64 158.89	14.43–14,392.77	Historical	**<0.001**
		Cortisol	219.96 ± 904.38 43.97	2.64–7,395.37		**0.002**
		Estradiol	1,207.23 ± 1,213.32 211.21	11.90–4,030.24		**<0.001**

Comparison column shows the statistical comparison (Kruskal–Wallis ANOVAs, Mann–Whitney *post hoc*) of the sample time periods’ hormone concentrations with the respective *P*-values (bolded *P*-values indicate significant differences (*P* < 0.05) between sample time periods). Note: archaeological samples include walruses of *n* = 1 female, *n* = 5 males and *n* = 47 unknown sex. Historical samples include walruses of *n* = 72 females, *n* = 71 males and *n* = 12 unknown sex. Modern samples include walruses of *n* = 21 females, *n* = 51 males and *n* = 1 unknown sex.

**Table 2:** Percent contribution to the dissimilarities in walrus bone steroid hormone concentrations (i.e. progesterone, testosterone, cortisol and estradiol) among sample time periods (archaeological, historical, modern)

		Percent (%) contribution	
Time period comparison	Average dissimilarity	Progesterone	Testosterone	Cortisol	Estradiol
Archaeological: historical	73	37	15	4	44
Modern: historical	76	36	15	5	44
Modern: archaeological	64	19	14	8	59

Average % dissimilarity is based on a scale of 0% = similar to 100% = no similarities. For example, archaeological and historical time periods had significantly different hormone concentrations (Table 1), and from this analysis, we see that the differences in progesterone (37%) and estradiol (44%) contributed the most to their dissimilarity (73%) in hormone concentrations compared to cortisol (only 4%).

When comparing steroid hormone concentrations separately among decades (1880s–2010s, excluding 1910s, 1920s and 1940s) with samples of all sexes, including unknowns, all steroid hormone concentrations were significantly different among decades (Kruskal–Wallis ANOVAs, *P* < 0.001 for all hormones; Table 3A–D). For progesterone, walruses collected in the 2010s were significantly lower than during the 1930s–1990s but similar to other decades (Table 3A). For testosterone, walruses from the 2010s were significantly lower than during the 1920s–1970s but similar to other decades (Table 3B). For cortisol, the 2010s were similar to all decades, except for the 1950s and 1960s, which were significantly higher (Table 3C). Estradiol concentrations in the 2010s were similar to other decades, except for the 1930s–1970s, which were significantly higher (Table 3D).

**Figure 2:**
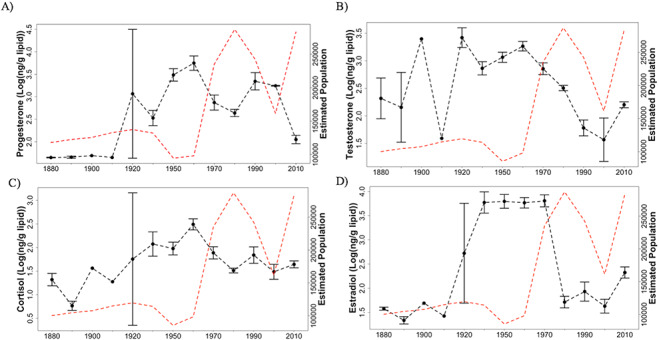
**A–D**. Median log-transformed steroid hormone concentrations of each decade ±1 SE for all walrus bones sampled between 1880s–2010s for progesterone (A), testosterone (B), cortisol (C) and estradiol (D) as black points. Samples were unavailable for the 1940s. Some decades only have one sample (1900s and 1910s) and only one datum plotted without error bars. Mean walrus population estimates are plotted as a broken red line; data provided in Supplementary S4. Significant differences (*P* < 0.05) in steroid hormone concentrations among decades are provided in Table 3A–D.

Overall, all steroid hormones followed a similar pattern, where median hormone concentrations were low in the 1880s and persisted with similar concentrations until the 1920s for testosterone, 1930s for progesterone and estradiol and 1950s for cortisol, when all hormone concentrations increased significantly (Table 3A–D; Fig. 2A–D). All steroid hormone concentrations increased until reaching peak median concentrations in the 1960s, except estradiol, which peaked in the 1970s (Table 4; Fig. 2A–D). Median hormone concentrations started to decrease in the 1970s but not significantly across all steroid hormones until the 1980s (Table 3A–D; Fig. 2A–D). Only median progesterone concentrations significantly increased in the 1990s before significantly decreasing again in the 2010s (Table 3A and Fig. 2A). Focusing on decades with adequate sample sizes (excluding 2000s, *n* = 2) and significantly higher steroid hormone concentrations compared with modern samples from the 2010s (Table 3A–D), progesterone and estradiol contributed most to the dissimilarities among decades (SIMPER; Table 5). Cortisol contributed < 10% to differences among decades.

### Steroid hormone concentration correlations with walrus population estimates

Correlation of steroid hormone concentrations with minimum, mean and maximum walrus population estimates were evaluated for samples collected from the 1960s through 2010s for all sexes (including unknowns). Progesterone and testosterone concentrations from all walrus samples were not significantly correlated with minimum walrus population estimates (*r* = −0.08, progesterone, *r* = −0.31, testosterone; Table 6). Cortisol and estradiol had significant negative correlations with minimum walrus population estimates (*r* = −0.31, cortisol, *r* = −0.07, estradiol; Table 6). Significant negative correlations were found among all steroid hormone concentrations with mean (*r* = −0.47, −0.45, −0.45, −0.49, progesterone, testosterone, cortisol and estradiol, respectively; Table 6) and maximum (*r* = −0.59, −0.60, −0.44, −0.44, progesterone, testosterone, cortisol and estradiol, respectively; Table 6) walrus population estimates.

**Table 3:** **A-D**.

(A) Progesterone										
	1880	1890	1920	1930	1950	1960	1970	1980	1990	2000
1890	0.99	-	-	-	-	-	-	-	-	-
1920	0.11	0.18	-	-	-	-	-	-	-	-
1930	**0.03**	0.14	0.79	-	-	-	-	-	-	-
1950	**<0.001**	**0.02**	0.55	0.05	-	-	-	-	-	-
1960	**<0.001**	**0.01**	0.39	**0.01**	0.44	-	-	-	-	-
1970	**0.01**	0.09	0.89	0.76	**0.04**	**0.004**	-	-	-	-
1980	**0.02**	0.14	0.74	0.91	**0.01**	**0.001**	0.62	-	-	-
1990	**<0.001**	**0.01**	0.36	**0.03**	0.47	0.83	**0.03**	**0.01**	-	-
2000	**0.03**	0.07	0.65	0.39	0.99	0.81	0.45	0.35	0.74	-
2010	0.38	0.58	0.19	**0.02**	**<0.001**	**<0.001**	**<0.001**	**0.003**	**<0.001**	0.05
(B) Testosterone										
	1880	1890	1920	1930	1950	1960	1970	1980	1990	2000
1890	0.57	-	-	-	-	-	-	-	-	-
1920	0.08	0.05	-	-	-	-	-	-	-	-
1930	0.10	0.08	0.42	-	-	-	-	-	-	-
1950	0.10	0.08	0.35	0.84	-	-	-	-	-	-
1960	**0.02**	**0.03**	0.62	0.42	0.19	-	-	-	-	-
1970	0.26	0.16	0.21	0.36	0.36	**0.03**	-	-	-	-
1980	0.93	0.56	**0.04**	**0.01**	**0.00**	**<0.001**	**0.03**	-	-	-
1990	**0.04**	0.39	**<0.001**	**<0.001**	**<0.001**	**<0.001**	**<0.001**	**0.003**	-	-
2000	0.13	0.43	**0.01**	**0.01**	**0.01**	**0.001**	**0.01**	0.10	0.88	-
2010	0.27	0.96	**0.01**	**<0.001**	**<0.001**	**<0.001**	**<0.001**	0.05	0.06	0.30
1890	0.50	-	-	-	-	-	-	-	-	-
1920	0.29	0.15	-	-	-	-	-	-	-	-
1930	**0.01**	**0.01**	0.60	-	-	-	-	-	-	-
1950	**0.005**	**0.009**	0.53	0.84	-	-	-	-	-	-
1960	**<0.001**	**<0.001**	0.19	0.09	0.05	-	-	-	-	-
1970	**0.02**	**0.02**	0.72	0.70	0.45	**0.01**	-	-	-	-
1980	0.37	0.17	0.54	**0.01**	**<0.001**	**<0.001**	**0.01**	-	-	-
1990	**0.04**	**0.03**	0.78	0.67	0.50	**0.04**	0.90	0.07	-	-
2000	0.66	0.35	0.60	0.23	0.18	**0.04**	0.28	0.92	0.34	-
2010	0.12	0.07	0.81	0.06	**0.003**	**<0.001**	0.05	0.23	0.24	0.63
(D) Estradiol										
	1880	1890	1920	1930	1950	1960	1970	1980	1990	2000
1890	0.65	-	-	-	-	-	-	-	-	-
1920	0.25	0.18	-	-	-	-	-	-	-	-
1930	**0.004**	**0.01**	0.45	-	-	-	-	-	-	-
1950	**<0.001**	**0.003**	0.25	0.42	-	-	-	-	-	-
1960	**<0.001**	**0.003**	0.26	0.45	0.93	-	-	-	-	-
1970	**<0.001**	**0.001**	0.16	0.17	0.46	0.40	-	-	-	-
1980	0.41	0.29	0.45	**<0.001**	**<0.001**	**<0.001**	**<0.001**	-	-	-
1990	0.64	0.41	0.36	**0.002**	**<0.001**	**<0.001**	**<0.001**	0.68	-	-
2000	0.86	0.60	0.42	0.07	**0.02**	**0.03**	**0.01**	0.73	0.90	-
2010	0.33	0.25	0.48	**<0.001**	**<0.001**	**<0.001**	**<0.001**	0.86	0.57	0.68

*P*-values from Dunn’s pairwise *post hoc* tests for individual hormones progesterone (A), testosterone (B), cortisol (C) and estradiol (D) by decade 1880s - 2010s (excluding 1900s and 1910s, n = 1 for each decade). Bolded *P*-values indicate significant differences (*P* < 0.05) among decadal median hormone concentrations presented in Table 4. Note: no specimens available for 1940s.

Spearman’s rank correlations were also performed with hormones measured in females and walrus population estimates. The correlations of female hormones to the minimum walrus population estimates were only significant for estradiol concentrations (*r* = −0.26; Table 6). Significant negative correlations were determined among female walrus progesterone, cortisol and estradiol concentrations with mean (*r* = −0.49, −0.46, −0.54, respectively; Table 6) and maximum population estimates (*r* = −0.68, −0.47, −0.57, respectively; Table 6). Female testosterone also exhibited a significant negative correlation with mean estimates (*r* = −0.25; Table 6) and the maximum population estimates (*r* = −0.55; Table 6). Based on these results that steroid hormones exhibited consistent significant negative correlations with mean walrus population estimates (Table 6), we discuss only correlations with mean population estimates.

### Steroid hormone concentration correlations with Chukchi Sea ice cover

**Figure 3:**
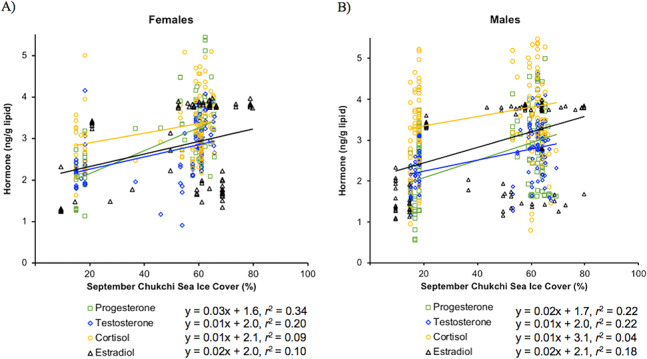
**A and B**. Linear regression analyses of log-transformed progesterone (open green squares), testosterone (open blue diamonds), estradiol (open black triangles) and Box–Cox transformed cortisol (open orange circles) concentrations (ng/g lipid) with percent September Chukchi Sea ice cover for female (A) and male (B) walruses. All linear regressions were significant (*P* < 0.05). Thus, equations from linear regressions and respective *r^2^* values are provided for each steroid hormone.

Female steroid hormones had significant positive relationships with September sea ice cover (*r^2^* = 0.34, 0.20, 0.09, 0.10, progesterone, testosterone, cortisol and estradiol, respectively, *P* = < 0.001, progesterone and testosterone, *P* = 0.003, cortisol, and *P* = 0.002, estradiol; Fig. 3A). Similar to females, all male steroid hormones had a significant positive relationship with September sea ice cover (*r^2^* = 0.22, 0.22, 0.04, 0.18, progesterone, testosterone, cortisol and estradiol, respectively, *P* = < 0.001 progesterone, testosterone and estradiol, *P* = 0.02, cortisol; Fig. 3B).

### Steroid hormone concentrations between sexes

**Figure 4:**
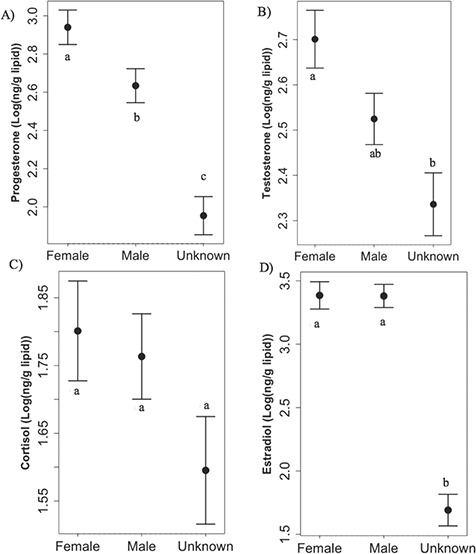
**A–D.** Log-transformed median ± 1 SE steroid hormone concentrations of all walrus bone samples plotted by sex for progesterone (A), testosterone (B), cortisol (C) and estradiol (D). Different letters denote significant differences (Kruskal–Wallis, Mann–Whitney, *P* < 0.05). Note: graphs are not on the same scale. Sample sizes were *n* = 94, *n* = 127 and *n* = 60 for females, males and unknowns, respectively.

As expected, female walruses had significantly higher progesterone steroid hormone concentrations compared with males (Kruskal–Wallis ANOVA, Mann–Whitney, *P* = 0.004, Fig. 4A), but females and males had similar testosterone concentrations (Kruskal–Wallis ANOVA, Mann–Whitney, *P* = 0.06; Fig. 4B). Females had significantly higher progesterone and testosterone concentrations compared to individuals of unknown sex (Kruskal–Wallis ANOVA, Mann–Whitney, *P* = < 0.001, 0.005, respectively), when all samples were pooled across all time periods (Fig. 4A–B). Males also had significantly higher progesterone concentrations compared to walruses of unknown sex (Kruskal–Wallis ANOVA, Mann–Whitney, *P* = 0.01, Fig. 4A), but testosterone concentrations were not statistically different (*P* = 0.35; Fig. 4B). Males, females and unknowns had similar cortisol concentrations (Kruskal–Wallis ANOVA, *P* = 0.26; Fig. 4C). Finally, both males and females had similar and significantly higher estradiol concentrations compared with individuals of unknown sex (Kruskal–Wallis ANOVA, Mann–Whitney, *P* = < 0.001, both sexes; Fig. 4D). Assigning the correct sex to known male and female walruses based on LDA of their progesterone, testosterone and cortisol concentrations was not successful based on relatively low matching success (LDA, 56% jackknife adjusted matching success); therefore, assigning sex to unknown samples based on bone hormone concentrations was not possible in this study. Overlapping ranges in steroid hormone concentrations among age classes of different sexes adds to the difficulty in assigning sex to unknown individuals solely based on these steroid hormone concentrations (Table 7).

## Discussion

This is the first application of measuring steroid hormones in bone as a tool for understanding the physiological response of a marine mammal to environmental and population changes. Specifically, reproductive and stress-related hormones were extracted from walrus bones collected over the past three millennia to determine how reproductive activity and stress (as indicated by biomarker cortisol) in walruses relate to changes in population and sea ice extent estimates. Overall, we provide evidence that bone is a valuable matrix for measuring steroid hormones that are indicators of walrus physiology and correlate with changes in the environment, as well as walrus population estimates.

**Table 4:** Total walrus sample sizes and sample size by sex with mean and median steroid hormone concentrations ±1 SD (ng/g lipid) among decades 1880–2010Decades with less than three total samples do not have a reported median value.

					Mean ± 1 SD Median			
Decade	Total Sample Size (*n*)	Females (*n*)	Males (*n*)	Unknown (*n*)	Progesterone (ng/g lipid)	Testosterone (ng/g lipid)	Cortisol (ng/g lipid)	Estradiol (ng/g lipid)
1880	5	0	1	4	44.77 ± 2.03 44.65	1,751.02 ± 3,328.63 207.61	23.93 ± 17.40 20.88	36.80 ± 6.10 37.84
1890	2	0	2	0	45.47 ± 3.39	324.37 ± 411.78	5.97 ± 1.86	22.09 ± 5.42
1900	1	0	0	1	49.20	2497.51	36.46	49.20
1910	1	0	1	0	44.78	38.98	18.85	26.70
1920	2	0	1	1	15,772.94 ± 2,245.35	2,860.95 ± 1,585.51	724.66 ± 1,021.65	2,867.61 ± 3,986.04
1930	13	8	3	2	1,233.77 ± 1,608.23 337.25	2,057.57 ± 2,929.38 731.29	645.96 ± 943.90 118.65	4,988.41 ± 2,215.84 5,894.00
1950	31	17	14	0	5,277.13 ± 7,213.90 3063.16	1,619.71 ± 1,633.64 1160.74	636.41 ± 1,841.25 95.26	5,395.96 ± 2,651.48 6,225.74
1960	36	10	23	3	30,552.71 ± 6,6730.32 6266.90	3,871.16 ± 7,283.70 1845.62	1,049.13 ± 1,656.01 311.37	5,428.12 ± 2,517.62 5,819.41
1970	27	7	19	1	6,880.16 ± 19,803.75 743.85	1,668.41 ± 2,691.81 716.60	654.80 ± 2,099.75 77.21	6,039.13 ± 1,946.08 6,385.93
1980	25	22	3	0	825.99 ± 801.14 435.49	395.84 ± 285.91 320.01	41.31 ± 29.33 32.50	550.44 ± 1,696.07 51.72
1990	10	6	4	0	9,056.92 ± 11,558.91 2209.00	81.78 ± 77.58 61.24	176.87 ± 208.44 83.95	241.80 ± 419.49 85.39
2000	2	2	0	0	1,759.15 ± 50.69	53.16 ± 54.15	32.63 ± 16.08	44.95 ± 20.16
2010	73	21	51	1	440.12 ± 845.23 112.38	443.99 ± 1,677.64 158.89	219.96 ± 904.38 43.97	1,207.23 ± 1,213.32 211.21

**Table 5:** Percent contribution to the dissimilarities in walrus bone steroid hormone concentrations (i.e. progesterone, testosterone, cortisol and estradiol) among decades that are significantly different (*P* < 0.05) from the 2010s with sample size *n* ≥ 10

		Percent (%) contribution
Decade comparison	Average dissimilarity	Progesterone	Testosterone	Cortisol	Estradiol
1930: 2010	73	12	23	7	58
1950: 2010	76	35	13	6	47
1960: 2010	80	45	14	6	35
1970: 2010	74	22	11	3	64
1980: 2010	69	38	16	5	41
1990: 2010	84	72	5	4	19

Average % dissimilarity is based on a scale of 0% = similar and 100% = no similarities. For example, of all the significant differences measured in hormones among the 1960 and 2010 time periods, changes in cortisol only contributed 6% to the overall dissimilarity in hormone concentrations. Whereas, changes in progesterone (45%) and estradiol (35%) during the same time period contributed the most to the measured dissimilarity among these decades. Thus, we can infer that changes in progesterone and estradiol are the main drivers of hormone differences among these decades.

**Table 6:** Spearman’s rank correlation of bone steroid hormones compared with the Pacific walrus population estimates (min, mean, max, see Supplementary S4)

Hormone	*r*-value (min, mean, max)	*P*-value (min, mean, max)	*r*-value (females only, min, mean, max)	*P*-value (females only, min, mean, max)
Progesterone	−0.08, **−0.47**, **−0.59**	0.29, **< 0.001**, **< 0.001**	−0.02, **−0.49**, **−0.68**	0.89, **< 0.001**, **< 0.001**
Testosterone	−0.31, **−0.45, −0.60**	0.05, **< 0.001, < 0.001**	−0.08, **−0.25, −0.55**	0.52, **0.04**, **< 0.001**
Cortisol	**−0.31, −0.45, −0.44**	**<0.0001, < 0.001, < 0.001**	**−0.29, −0.46, −0.47**	**0.01, < 0.001, < 0.001**
Estradiol	**−0.07, −0.49, −0.44**	**<0.001, < 0.001, < 0.001**	**−0.26, −0.54, −0.57**	**0.03, < 0.001, < 0.001**

Significant *P*-values (*P* < 0.05) and *r*-values are bolded. Male (*n* = 100), female (*n* = 68) and unknown (*n* = 5) walruses were pooled, but females (*n* = 68) were also run separately.

### Stress-related response of walruses to changes in sea ice and population estimates

One of our main objectives was to address bone cortisol concentrations of the modern walrus population with respect to the current changing climate in the Arctic and the lack of a summer sea ice platform. Our results show that the modern walrus population has similar bone cortisol concentrations to the archaeological population indicating a possible physiological resilience to current Arctic conditions (Table 1). The industrial revolution is generally agreed to have started in 1750 CE (IPCC, 2013), and emissions from North America and Europe did not reach the Arctic until after 1860s (McConnell and Edwards, 2008). While the Industrial Revolution is attributed to the start of anthropogenic emissions of CO_2_ (IPCC, 2013), the rapid rate of climate change in the Arctic was not described until the 1950s (Kaufman *et al.*, 2009; Kinnard *et al.*, 2011). Our archaeological samples are older than 0 BP (i.e. 1950 CE; Supplementary S1). Therefore, our archaeological dataset is as close to a baseline walrus population as possible and provides a reasonable control group in our study. Modern samples are from 2014 to 2016 CE and represent a population that could be expected to exhibit a stress response due to a reduction of summer sea ice over the past ~13 years (Jay *et al.*, 2012; MacCracken, 2012). Since 2007, up to 35 000 walruses have hauled out on the beach near Point Lay instead of utilizing sea ice haulouts, possibly a response to climate change (Jay *et al.*, 2012). The potential stressors due to lack of sea ice in the Chukchi Sea include longer foraging trips for calves and females that have high energy demands (Jay *et al.*, 2012; Noren *et al.*, 2014), decreases in calf survival due to human and natural-induced stampedes (Jay *et al.*, 2012; Udevitz *et al.*, 2013), predicted depleted benthic food sources (Bluhm and Gradinger, 2008), reduced foraging activity (Jay *et al.*, 2017), potentially increased encounters with polar bears (*Ursus maritimus*), and the introduction of novel diseases to the population (Burek *et al.*, 2008; Garlich-Miller *et al.*, 2011). However, our results show that walruses do not have significantly increased cortisol concentrations over the past 9 years compared with our control samples (i.e. archaeological bone), and cortisol concentrations contributed < 10% to the differences in steroid hormone concentrations among decades and archaeological, historical and modern time periods (Tables 1, 2 and 5).

While both females and males had a significant positive correlation of cortisol with the 15-year average of sea ice cover, the *r*^2^ values were low for both sexes (*r^2^* = 0.09 and 0.06, respectively), most likely due to high variability among the correlations of steroid hormones and sea ice cover (Fig. 3A–B). During September, sea ice cover is generally reaching its minimum extent, and reproductively active females are present in the Chukchi Sea, making sea ice a crucial foraging platform during this time (Fay, 1982). Thus, if the lack of sea ice was a chronic stressor, female cortisol concentrations should show a strong negative correlation with sea ice cover. As male walruses typically spend their summers in Bristol Bay, AK or along Russian coastlines, with less reliance on Chukchi sea ice in September (Fay, 1982), we anticipated that sea ice would not have a strong relationship with male cortisol concentrations. Walruses have experienced previous warming and cooling periods in the Arctic that have included periods of contractions and extensions of sea ice (Dyke *et al.*, 1999; Garlich-Miller *et al.*, 2011), apparently with no significant change in trophic position over the past 4000 years (Clark *et al.*, 2019). Current warming trends and sea ice reduction, however, are occurring at unparalleled rates and trophic changes may not yet be detected in bone stable isotopes (Clark *et al.*, 2019). The same caveat applies to our cortisol data (discussed below) because walrus cortical bone, conservatively, represents a 10–20 year accumulated average hormone signature (Charapata *et al.*, 2018), and any chronic stress response to the rapid and recent changes in sea ice may not yet be detected in the bone. There is high genetic diversity within the walrus population providing potential resiliency to many environmental changes (Sonsthagen *et al.*, 2012; Shitova *et al.*, 2017). Therefore, it is not necessarily unexpected that the modern walrus population has similar cortisol concentrations to the archaeological population or that cortisol in bone is weakly related to September Chukchi sea ice cover (Table 1; Fig. 3A–B), supporting the possibility that walruses have coping strategies for current receding sea ice.

**Table 7:** Mean and median steroid hormone concentrations (i.e. progesterone, testosterone, cortisol and estradiol) ± 1 SD and concentration ranges (min and max) (ng/g lipid) for all walrus bone samples by sex and age class

Sex	Age class	Sample size *(n)*	Hormone	Mean ± 1 SD (ng/g lipid)	Median (ng/g lipid)	Range: min–max (ng/g lipid)
			Progesterone	10,747.83 ± 44,116.82	599.54	13.47–276,407.72
	Adult	74	Testosterone	1,012.14 ± 2,254.20	313.74	8.09–14,392.77
			Cortisol	531.42 ± 1,647.76	54.06	6.43–10,062.76
			Estradiol	2,525.52 ± 2,843.98	364.43	17.41–7,455.46
			Progesterone	7,026.38 ± 7,626.14	6,261.61	18.36–30,329.86
Female	Subadult	18	Testosterone	2,660.09 ± 2,278.47	2,497.74	178.61–7,621.84
			Cortisol	968.19 ± 2,440.12	158.51	32.10–10,412.57
			Estradiol	5,982.63 ± 2,959.51	6,523.68	18.36–9,460.71
			Progesterone	344.53 ± 136.00	-	248.37–440.70
	Unknown	2	Testosterone	651.26 ± 723.34	-	139.78–1,162.74
			Cortisol	39.33 ± 27.85	-	19.64–59.02
			Estradiol	2,925.38 ± 1,195.26	-	2,080.2–3,770.56
			Progesterone	2,662.02 ± 7,641.24	296.95	3.49–42,873.22
	Adult	90	Testosterone	1,039.56 ± 4,445.00	224.90	14.43–41,718.13
			Cortisol	253.97 ± 775.51	43.41	2.24–6,226.90
			Estradiol	2,671.68 ± 2,728.38	2,256.50	11.90–8,329.49
			Progesterone	7,227.34 ± 17,787.85	1,682.18	15.33–98,533.53
Male	Subadult	35	Testosterone	1,760.64 ± 2,821.26	674.84	22.09–12,644.41
			Estradiol	4,376.77 ± 2,778.93	5,685.38	14.46–9,754.42
			Progesterone	124.87 ± 141.93	-	24.52–225.23
	Unknown	2	Testosterone	324.42 ± 96.94	-	255.88–392.97
			Cortisol	225.52 ± 79.03	-	169.63–281.40
			Estradiol	1,131.40 ± 1,557.96	-	29.76–2,233.05
			Progesterone	32,378.01 ± 68,611.29	485.12	43.75–185,780.36
	Adult	7	Testosterone	3,574.31 ± 5,806.95	851.46	207.09–16,379.21
			Cortisol	1,023.05 ± 1,793.44	112.01	9.6–4,910.70
			Estradiol	2,955.13 ± 2,767.81	4,196.48	30.35–5,686.17
			Progesterone	1,514.88 ± 3,053.47	101.24	45.08–9,230.67
Unknown	Subadult	9	Testosterone	2,136.43 ± 2,653.61	365.27	44.94–7,694.14
			Cortisol	240.92 ± 592.73	29.61	3.76–1,816.75
			Estradiol	2,984.46 ± 2,930.28	3,948.18	38.04–7230.73
			Progesterone	183.17 ± 289.06	87.38	15.09–1,390.73
	Unknown	44	Testosterone	268.91 ± 309.39	198.73	35.61–1,803.85
			Cortisol	141.50 ± 323.08	39.39	13.70–1,889.60
			Estradiol	953.63 ± 1,851.33	44.04	10.01–7161.93

Walruses exhibited an increase in cortisol concentrations in the 1950s and1960s (and possibly 1970s, *P* = 0.05) compared with modern-day walruses (Table 3C; Fig. 2C). The most plausible explanation for the elevated cortisol concentrations is the exponential increase of the walrus population during the 1950s–1970s (Fay *et al.*, 1989, 1997; Garlich-Miller *et al.*, 2011; Taylor and Udevitz, 2015; Taylor *et al.*, 2018). This is further supported by the significant negative correlations of cortisol measured in female walruses, as well as pooled samples, with population estimates (Table 6), because the population was increasing at this time (1950s–1970s), but was lower compared to their carrying capacity reached around 1975–1980 (Taylor and Udevitz, 2015; Taylor *et al.*, 2018). Highly fecund females may have higher cortisol concentrations (Gardiner and Hall, 1997) because of the need for increased energy stores (Noren *et al.*, 2014), carrying a fetus (Hunt *et al.*, 2014), physically giving birth, caring for the calf after birth, including lactation, and protecting their calf from predators and other dangers (Fay, 1982). Reproductively active males could have higher cortisol concentrations (Bartsh *et al.*, 1992) during this fecund period due to competition with other males for reproductive females (Fay, 1982). Thus, the increased cortisol concentrations observed in walrus bone from the 1950s to 1960s (possibly 1970s, *P* = 0.05; Table 3C) compared with the 2010s is likely due to this reproductively active 20-year time period (Fig. 2C; Table 3C).

Cortisol helps an animal cope with nutritional stress by metabolizing fat stores, conserving blood glucose levels and increasing circulating fatty acids (Norris, 1997; Peckett *et al.*, 2011; Kershaw and Hall, 2016). For example, in addition to the population increasing in the 1950s–1970s, competition for resources during this reproductive period (1950s—1970s) could contribute to increased cortisol concentrations (Hohmann *et al.*, 2009). However, clam populations did not exhibit signs of depletion in the walrus habitat until the 1970s (Lowry *et al.*, 1980), and the stress response to this depletion of resources and subsequent increase in competition measured in bone (via biomarker cortisol) would potentially not be detectable until the 1980s. Interestingly, walruses had significantly lower cortisol concentrations in the 1980s compared with animals from the 1960s (Table 3C). Walruses are highly adapted benthic feeders, whose primary prey are clams; however, upwards of 100 taxa have been identified in their stomachs (Fay, 1982; Sheffield and Grebmeier, 2009). Sheffield and Grebmeier (2009) found no significant difference in mollusk vs non-mollusk prey in stomachs of walruses during 1975–1985 providing evidence that decreases in clam populations in the 1970s would not necessarily result in nutritional stress for walruses. Further, walruses exhibited comparable general foraging locations and trophic positions based on δ^13^C and δ^15^N values from the 1970s and 1980s (Clark *et al.*, 2019). These previous studies support our findings that walruses were not nutritionally stressed in the 1980s.

### Reproductive activity of the walrus population in relation to population estimates and sea ice extent

Another objective of this study was to assess reproductive hormones (i.e. estradiol, progesterone and testosterone) as a tool to evaluate the reproductive status of the walrus population. With receding Arctic sea ice, walruses are utilizing terrestrial haulouts in the summer (Jay *et al.*, 2012, 2017) with predictions of a decrease in population carrying capacity (MacCracken, 2012; Taylor *et al.*, 2018) and calf survival (Udevitz *et al.*, 2013). We found negative correlations among reproductive hormones and mean walrus population estimates; progesterone and estradiol exhibited the strongest correlations (Table 6). Thus, when the walrus population is stable or high, possibly at carrying capacity, reproductive steroid hormone concentrations are low, and when population numbers are low, reproductive hormone concentrations are relatively high. These significant correlations complement our decadal differences in reproductive steroid hormones. When the population is low, but increasing (1950s–1970s), reproductive hormone concentrations in these decades are significantly higher compared with other decades, when the population is decreasing or stable (Fig. 2A, B and D).

The current walrus population has significantly lower median progesterone and estradiol concentrations compared with decades of known population increases (1950s–1970s; Fig. 2A and D; Table 4), indicating the modern walrus population is most likely at a relatively high number, possibly at carrying capacity. Other walrus reproductive data support our results of bone steroid hormones negatively correlating with mean population estimates and the modern walrus population possibly nearing carrying capacity. A USFWS preliminary 2014 walrus population evaluation (MacCracken *et al.*, 2017), based on genetic mark-recapture methods, was estimated at 283 213 walruses (Supplementary S4), a substantial increase from the biased low Speckman *et al.* (2011) estimate (mean = 129 000 animals), which supports our interpretation of negative correlations of reproductive hormones with population size, and the reproductive activity of modern walruses. An update to Taylor and Udevitz (2015) found that the walrus population underwent a lesser population decline from 1980s to present and that the population could have reached an equilibrium by 2015 (Taylor *et al.*, 2018). Further, the age of sexual maturity in females potentially plateaued in the late 2000s indicating that a walrus carrying capacity could have been reached by the late 2000s (Clark *et al.*, 2020). Overall, progesterone and estradiol concentrations are low in the modern walrus population (Table 1), lending more evidence to the population possibly producing fewer calves (Garlich-Miller *et al.*, 2006), and/or the population is nearing carrying capacity (Udevitz *et al.*, 2017; Clark *et al.*, 2020).

Reproductive steroid hormones exhibited significant positive correlations with 15-year (progesterone and testosterone) and yearly (estradiol) averages of sea ice, giving insight into walrus reproduction and its relationship to September sea ice extent (Fig. 3A–B). For females, progesterone had the highest correlation with Chukchi sea ice extent, followed by testosterone and estradiol (Fig. 3A). Progesterone in females would be a suitable biomarker of reproductive activity in relation to sea ice extent because progesterone is elevated for months, including September, throughout the 15-month walrus gestation (Fay, 1982; Kinoshita *et al.*, 2012; Triggs, 2013) resulting in the relatively stronger correlation to sea ice compared to estradiol and testosterone. When sea ice is not available, walruses tend to haul out on land, spend less time actively foraging and have less access to productive benthic foraging areas (Jay *et al.*, 2012, 2017). The lack of sea ice has been predicted to result in a 7–18% and 25–34% decrease in seasonal median body mass for non- and reproductively-active female walruses, respectively (Udevitz *et al.*, 2017). Udevitz *et al.* (2017) predicted that this decrease will not result in loss of females and their respective young over a season because this loss in body mass is common in pinnipeds and generally replaced in the winter. However, if sea ice continues to be absent during the summer and yearly blubber reserves cannot be replenished during the winter, the cumulative loss in mass over multiple years could result in reduced fecundity (Udevitz *et al.*, 2017). Our results support a similar idea with bone progesterone having a positive correlation with sea ice for female walruses (Fig. 3A). If walruses are subjected to decades of open water and reduced sea ice in the summer, female body condition could worsen resulting in lower reproductive activity and less progesterone deposited into bone. This is supported by the linear regression analysis for females; those that experienced < 50% sea ice cover (on average) over a 15-year period had lower progesterone concentrations (Fig. 3A).

Males and females followed a similar pattern of reproductive hormones and sea ice (Fig. 3A–B). Testosterone in males had a significant positive relationship with sea ice (*r^2^* = 0.22; Fig. 3B). Males typically do not rely on summer Chukchi sea ice (Fay, 1982) although they have been documented moving northward with females in the summer to the Chukchi Sea (Jay *et al.*, 2012). Spermatogenesis, initiated seasonally by testosterone in males (Muraco *et al.*, 2012), occurs from fall to early spring, with peak periods of spermatogenesis in the winter for adults and early spring for subadults (Fay, 1982). Therefore, it is likely male testosterone in bone is not directly related to September Chukchi sea ice extent (Fig. 3B). However, the positive correlation could be explained by potent males arriving earlier than females at their winter breeding grounds because females may stay longer in the Chukchi Sea due to later sea ice formation in the fall, resulting in less male reproductive activity (Jay *et al.*, 2012; Muraco *et al.*, 2012). Another possibility is subadult males express potency later in the year (spring) compared to adults (Fay, 1982). Possibly, this is an artifact of later sea ice formation and walrus reproductive timing paradigms or an adaptation to this potential mismatch in walrus reproductive timing due to changing Chukchi sea ice extent.

### Steroid hormones as a tool to assign sex to unknown individuals and reproductive hormones among sexes

LDA analysis was unable to predict sex of known-sex walruses using steroid hormone concentrations (assignment accuracy ~ 56%). Interestingly, walruses of unknown sex had significantly lower progesterone and estradiol concentrations compared to known female and male walruses (Fig. 4A and D). This is most likely due to the majority of unknown individuals being from the archaeological period (*n* = 47 of 60 from archaeological time period), a time that had significantly lower progesterone and estradiol concentrations compared to the historical time period (Table 1). The majority of the known males and females were collected in the historical time period (*n* = 72 of 94 females and *n* = 71 of 127 males), when all steroid hormones were significantly higher than archaeological and modern walruses (Table 1). Walruses of unknown sex are still valuable specimens, especially for archaeological time periods where available samples are bone fragments that cannot be adequately sexed based on morphometrics (e.g. Taylor *et al.*, 2020). This is the first attempt to sex walruses based on steroid hormones and walrus age class is likely an important predictor to be considered in these efforts. Future studies should explore sex assignment based on steroid hormones in bone that include hormonal profiles of juveniles, reproductively mature, and senescent males and females.

Reproductive hormones among walruses of known sex displayed some unexpected patterns. Females had the highest progesterone concentrations, as expected, but males also had relatively high progesterone concentrations (Table 7). This could be due to progesterone playing a role in male sexual behaviour (Wagner, 2006). Wagner (2006) suggested that during times of stress in male rats, circulating testosterone was inhibited, and progesterone may play a supplementary role in male sexual behaviour in response to the testosterone decrease. Another possibility for elevated progesterone concentrations in males is that progesterone is a precursor to testosterone, cortisol and estradiol (Koal *et al.*, 2012). Progesterone may be stored by male bones as a ‘backup’ hormone to metabolize other useful steroid hormones (e.g. testosterone) in case the animal cannot absorb or metabolize cholesterol into these important hormones (Simonen *et al.*, 2000). Interestingly, adult females had higher median testosterone concentrations compared to adult males (313.74 ± 2254.20 ng/g lipid, 224.90 ± 4450.00 ng/g lipid for females and males, respectively). For females, high testosterone in female grizzly bears (*Ursus arctos*) has been linked to social stressors including protecting cubs and resources from predators and conspecifics, in addition to reproductive activity (Bryan *et al.*, 2013). It is possible that the higher testosterone concentrations in females in this study were driven by the high testosterone concentrations during the 1950s–1970s, aiding in the population increase during those decades, and the subsequent social stressors associated with an increasing walrus population (competition and protection of calves; Fig. 2B). Estradiol concentrations were similar among males and females (Fig. 4D). High estradiol concentrations in females are an indicator of pregnancy (Charapata *et al.*, 2018) and possibly estrus (Larsen Tempel and Atkinson, 2020) but also play a role in adult male spermatogenesis (Hess, 2003; Carreau *et al.*, 2006). Additionally, estradiol plays a key role in maintaining bone mineral density and is potentially synthesized in the bone due to aromatization of testosterone (Yarrow *et al.*, 2010). This may lead to a shorter reservoir time (~1 year) of estradiol in bone compared to other steroid hormones (Charapata *et al.*, 2018). Despite these interesting patterns, reproductive hormones measured in bone are biomarkers of walrus reproductive activity based on their significant correlations with changes in population and sea ice cover estimates (Table 6; Fig. 3A–B).

### Data limitations

Measuring steroid hormones in bone to study physiology is a new approach, and we therefore acknowledge certain limitations. Radiocarbon analyses of archaeological marine mammal bone are not exact and are further complicated by the mixing of marine carbon known as the marine reservoir effect (Stuiver and Polach, 1977; Dumond and Griffin, 2002). Therefore, radiocarbon dates from terrestrial plant and mammal tissues associated with walrus bone from archaeological sites (see Clark *et al.*, 2019) are used to correct for the marine reservoir effect, resulting in a more reliable range of collection dates compared to directly radiocarbon dating the walrus bones.

The extraction efficiency of progesterone was 51%, and most likely contributed to the number of ND samples for progesterone (*n* = 35 of 281 total). Either using a stronger solvent (e.g. 2:1 chloroform: methanol) or performing a second methanol extraction may result in a higher extraction efficiency and result in lower number of ND samples for progesterone. Detectable progesterone in the majority of samples was corrected for the low extraction efficiency based on the added isotopically labelled internal standard prior to the hormone extraction (Charapata *et al.*, 2018).

Steroid hormones measured in walrus cortical bone most likely represent an accumulated average over the past 10–20 years of a walrus’s life, except estradiol (~1 year average; Charapata *et al.*, 2018). Thus, progesterone, testosterone and cortisol measured in modern walruses may not be representative of steroid hormones at time of sample collection (2014–2016) but an average hormone concentration from the past 10–20 years. Cortical bone is vascularized, allowing the transport of lipophilic steroid hormones in the blood from the gonads and adrenal cortex to the bone tissue, where they would potentially be deposited into the mineralized matrix or associate with bone cell membranes (Yarrow *et al.*, 2010; Usha and Nandeesh, 2012; During *et al.*, 2015). Thus, the hormone signal throughout the collection years (2014–2016) would still be integrated into the cortical bone. However, the long-term accumulated average is imperative when monitoring changes in physiology over extensive time periods because they are not skewed by acute stressors or reproductive events (Charapata *et al.*, 2018).

We used a variety of bone elements to measure hormones (Supplementary S1, 2 and 3). Charapata *et al.* (2018) found similar hormone concentrations (progesterone, testosterone, cortisol and estradiol) in paired walrus skull and mandible samples. This is in agreement with Yarrow *et al.* (2010), who found similar testosterone concentrations in paired tibias and fibias of rats. Thus, we assumed hormone concentrations were comparable among elements, but further studies are needed to confirm this is true among all skeletal elements. This type of method validation has been performed with stable isotopes of a variety of marine mammal species (Clark *et al.*, 2017; Smith *et al.*, 2020) and would be beneficial for future steroid hormones studies with bone.

### Conclusions and future studies

This is the first study to apply the method developed by Charapata *et al.* (2018) as a tool to monitor long-term changes in walrus physiology. Results showed modern walruses have similar cortisol concentrations to the archaeological walruses suggesting that current environmental conditions have not elevated stress-related hormone concentrations. September sea ice extent had a weak correlation to the stress biomarker cortisol, indicating the physiological stress response of walruses is not strongly associated with changes in sea ice extent. Future monitoring of cortisol in walrus bone may be warranted, however, to determine if the slow turnover rate in cortical bone could result in a mismatch between recent elevated circulating cortisol in response to low sea ice extent and the paired cortisol concentrations in cortical bone that reflect multiple years. Progesterone in female walruses was positively correlated with 15-year averages of sea ice extent, indicating higher female reproductive activity is associated with greater Chukchi Sea ice cover in September.

Our method of measuring bone steroid hormones is pertinent for management and conservation of walruses and potentially other marine mammals. Our results indicate walruses have not demonstrated an increase in the stress biomarker cortisol in response to recent reductions in sea ice. Walrus summer sea ice habitat, however, is expected to be gone as early as 2050 (IPCC, 2013), and other stressors are on the rise (e.g. ship traffic, possible chemical and other noise pollution). Thus, continued monitoring of cortisol and reproductive hormone concentrations in walruses is recommended. Currently, progesterone, testosterone and estradiol concentrations are low and similar to concentrations in archaeological samples. This, along with their negative correlations with mean walrus population estimates, potentially indicates low calf production and a population that may be approaching carrying capacity. It is important to note that the current carrying capacity may be lower relative to historical carrying capacity due to climate change related stressors (MacCracken, 2012; Clark *et al.*, 2020). Bone steroid hormones could also be useful for assessing physiological responses to climate change in other pagophilic marine mammals, including polar bears and ice seals. Museum collections, such as UAM and Smithsonian Institution National Museum of Natural History and other archived sample databases (e.g. stranding networks), house specimens to conduct similar studies on various marine and terrestrial mammal species.

## Funding

This work was supported by the National Science Foundation under Grant No. 1263848 with contributions from Bureau of Ocean Energy Management. Additional funding was provided by a University of Alaska Fairbanks Center for Global Change Student Research Grant with funds from the Cooperative Institute for Alaska Research and the Dr Donald Hood Memorial Scholarship for Marine Science.

## Supplementary Material

Appendix_coaa135
