# Supplementary material for: Steroid hormones in Pacific walrus bones collected over three millennia indicate physiological responses to changes in estimated population size and the environment
Source: Conserv Physiol. 2021 Jan 19;9(1):coaa135. doi: 10.1093/conphys/coaa135 (PMC7836870; doi:10.1093/conphys/coaa135)
Supplement: Appendix_coaa135 [file appendix_coaa135.docx]

**Appendix 1:** List of archaeological walrus specimens, defined as bones with ages greater than 200 years before present (BP), with their respective provenience and steroid hormone concentrations data. Samples were collected from various archaeological collections from the University of Alaska (UAM: ARCH), Sanak Island excavation (SANAK), and collections curated by Dr. A. Jensen at Ukpeaġvik Iñupiat Corporation (Utqiaġvik). ‘Year Collected’ dates were acquired from Clark *et al.*, (2019). Samples with “*” are aged based on the carbon dates from above and below their collection sites.

**Appendix 1 continued:**

**Appendix 2:** List of historical walrus bone samples, collected from 1880 – 2006, and their respective provenience and steroid hormone concentrations data (progesterone (P), testosterone (T), cortisol (C), and estradiol (E), ng/g lipid). Samples were collected from University of Alaska Museum (UAM: Mamm:) and the Smithsonian Institute (Smith: Mamm:) with respective catalog numbers noted in “Source” . The 15-year average percent September Chukchi Sea ice coverage (Chukchi SIC 15 yr [% Cover]) and average percent September Chukchi Sea ice coverage (Chukchi SIC [% Cover]) are listed for each sample. Provenience data including age class, sex, pregnancy status, and present/absence of offspring based on museum records curated by their respective sources.

**Appendix 2 continued:**

**Appendix 2 continued:**

**Appendix 3:** List of modern walrus samples, defined as bones collected from 2014 – 2016 with provenience and steroid hormone concentrations data (progesterone (P), testosterone (T), cortisol (C), and estradiol (E), ng/g lipid). Bones were collected in a collaborative effort between Alaskan subsistence hunters from Savoonga and Gambell, the Eskimo Walrus Commission, the U.S. Fish and Wildlife Service, and the Alaska Department of Fish and Game (ADFG), Utqiaġvik subsistence hunters, and the North Slope Borough Department of Wildlife Management. The majority of these bones were opportunistically collected by hunters in the field, thus, specific bone elements collected were unknown and depicted with “-“. The 15-year average percent September Chukchi Sea ice coverage (Chukchi SIC 15 yr [% Cover]) and average percent September Chukchi Sea ice coverage (Chukchi SIC [% Cover]) are listed for each sample.


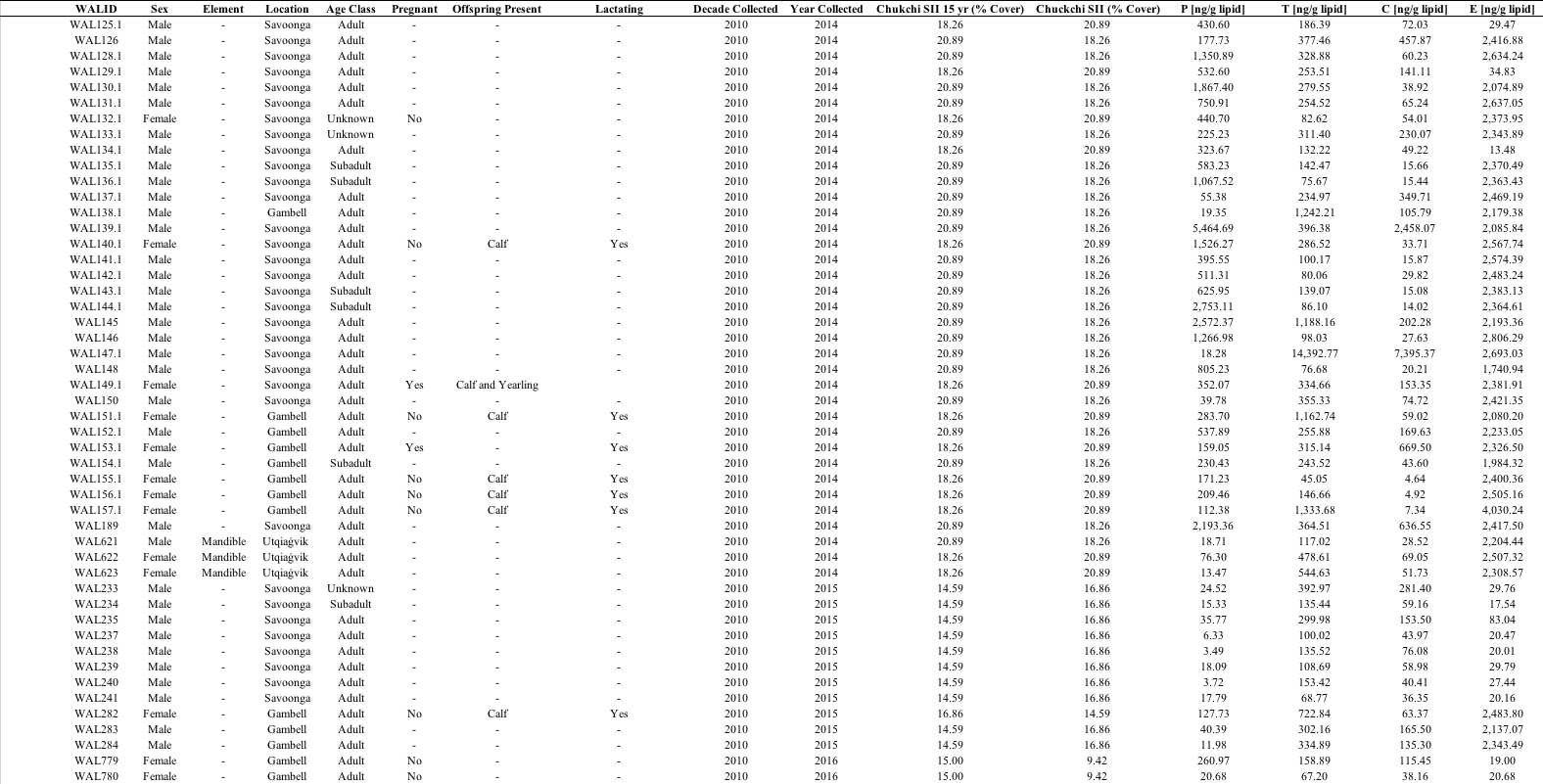


**Appendix 3 continued:**

**
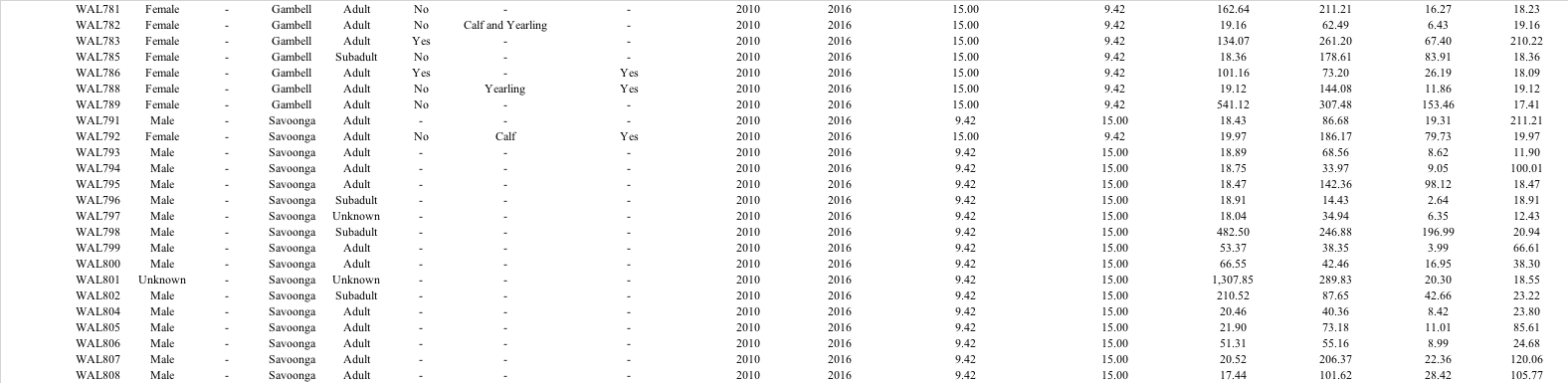
**

**Appendix 4**: Estimated minimum, mean, and maximum Pacific walrus populations from the published literature. Due to high variability in historical assessments based on different survey methods, all three population estimates were used for steroid hormone correlations. MacCracken *et al.*, (2017) preliminary estimate was used as the most recent, published estimate for 2014 – 2016.
